# The Discharge of Tuberculous Soldiers from the French Army

**Published:** 1915-07-03

**Authors:** 


### The Discharge of Tuberculous Soldiers from the French Army.

Professor Landouzy, Dean of the Paris Faculty of Medicine, recently presented to the permanent Commission on Tuberculosis an opportune report on the above subject. According to an account of the lecture given by the Paris correspondent of the official organ of the American Medical Association, the Professor believes that the years 1915 and 1916 are going to send out over the country 20,000 tuberculous subjects to scatter the germs of the disease in the districts in which they settle after their discharge from the Army. This regrettable state of affairs could, he thinks, be modified by radical measures on the part of the War Department. In the first place, it is indispensable to eliminate every man who is clearly affected with tuberculosis. In the next place, tuberculosis should not be, as at present, the disease to which is most seldom applied the form of discharge which permits a temporary or permanent pension. While there is reason to believe that tuberculosis is rarely contracted in barracks, and that military life is seldom the source of infection, there can be no doubt that the overwhelming exertion inseparable from this life is a factor in causing the latent disease to become active. At the present time the danger is increased by the fact that the *conseils de révision* have just placed on active service men who were previously discharged because of tuberculosis, but who are now found in good condition, and may again become a source of danger owing to the life of the trenches. Professor Landouzy asks whether, when these men become unmistakably tuberculous and incapable of taking part in the campaign, the Army has the right simply to rid itself of them by discharging them, and taking no further interest in them. He asserts, and very properly, that these men discharged on account of tuberculosis ought not to leave the Army without being assured of treatment and assistance. Such measures would tend to protect the general population from carriers of the disease. After the war there will be tuberculous patients who will be war invalids just as much as are the soldiers wounded in battle, and the nation will owe an equal debt of gratitude to each.

